# Plant responses to geminivirus infection: guardians of the plant immunity

**DOI:** 10.1186/s12985-021-01612-1

**Published:** 2021-07-09

**Authors:** Neha Gupta, Kishorekumar Reddy, Dhriti Bhattacharyya, Supriya Chakraborty✉

**Affiliations:** Molecular Virology Laboratory, School of Life Sciences, Jawaharlal Nehru University, New Delhi, 110067 India

**Keywords:** Autophagy, Begomovirus, Betasatellite, Defence, Geminivirus, Immunity, Pathogenesis, Resistance

## Abstract

**Background:**

Geminiviruses are circular, single-stranded viruses responsible for enormous crop loss worldwide. Rapid expansion of geminivirus diversity outweighs the continuous effort to control its spread. Geminiviruses channelize the host cell machinery in their favour by manipulating the gene expression, cell signalling, protein turnover, and metabolic reprogramming of plants. As a response to viral infection, plants have evolved to deploy various strategies to subvert the virus invasion and reinstate cellular homeostasis.

**Main body:**

Numerous reports exploring various aspects of plant-geminivirus interaction portray the subtlety and flexibility of the host–pathogen dynamics. To leverage this pool of knowledge towards raising antiviral resistance in host plants, a comprehensive account of plant’s defence response against geminiviruses is required. This review discusses the current knowledge of plant’s antiviral responses exerted to geminivirus in the light of resistance mechanisms and the innate genetic factors contributing to the defence. We have revisited the defence pathways involving transcriptional and post-transcriptional gene silencing, ubiquitin-proteasomal degradation pathway, protein kinase signalling cascades, autophagy, and hypersensitive responses. In addition, geminivirus-induced phytohormonal fluctuations, the subsequent alterations in primary and secondary metabolites, and their impact on pathogenesis along with the recent advancements of CRISPR-Cas9 technique in generating the geminivirus resistance in plants have been discussed.

**Conclusions:**

Considering the rapid development in the field of plant-virus interaction, this review provides a timely and comprehensive account of molecular nuances that define the course of geminivirus infection and can be exploited in generating virus-resistant plants to control global agricultural damage.

## Background

Geminiviruses belong to the largest family of plant viruses, *Geminiviridae*. Characterized by the circular, single-stranded DNA genome, they cause devastating diseases in plants, faring as one prominent reasons of global crop loss and compromised food security. Geminiviruses are phloem limited viruses and are transmitted by hemipterous insect vectors. Their unique virion includes a twinned icosahedral structure enclosing the circular genomic DNA. Replication of the DNA occurs through the rolling circle and recombination dependent mechanism [1]. In differentiated host cells, geminiviruses reprogram the cell cycle and transcriptional events [2], making the microenvironment suitable for its own replication. Inside the infected plant cell, host DNA polymerases convert the viral single-stranded DNA (ssDNA) into double-stranded DNA (dsDNA) and host nucleosomes pack the dsDNA forming minichromosomes that reside in host nucleus and can act as a template for virus transcription [3, 4]. Early transcription events drive the genes essential for virus replication and transcription, followed by late genes required for encapsidation and movement. By altering the host gene expression profile and regulating the host cell signalling pathways, geminiviruses induce severe diseases in plants which manifest as leaf curling, veinal swelling, chlorosis, growth stunting, stem bending, and smalling of leaves etc. [5, 6].

Based on their phylogenetic relationships, genome organization, host range and insect vectors, geminiviruses are categorized into nine genera- *Becurtovirus, Begomovirus, Capulavirus*, *Curtovirus*, *Eragrovirus, Grablovirus, Mastrevirus, Topocuvirus* and *Turncurtovirus* [7]. Among these, begomovirus constitutes the largest genus that are predominantly transmitted by whitefly [*Bemisia tabaci* Genn.] vector. While majority of the classified genera comprises a monopartite genome, begomoviruses can contain either monopartite or bipartite genome. Based on their geographical distributions and genetic diversities, begomoviruses are grouped into Old world (Africa, Europe, Australia, and Asia) and New World categories (America) [8]. The New World begomoviruses mostly have bipartite genome while the Old World ones contain both mono and bipartite genomes. The genome of a bipartite begomovirus contains two separately encapsidated DNA molecules, known as DNA-A and DNA-B, of sizes ranging from 2600 to 2800 nt [8]. Monopartite begomoviruses have genome of one DNA molecule which is structurally and genetically similar to DNA-A of bipartite begomoviruses*.* Both DNA-A and DNA-B include a common region (CR) of 200–250 nucleotides that encompasses a conserved stem-loop structure and the sequence TAATATTAC. The DNA-A component contains open reading frames (ORFs) encoding five to seven proteins while DNA-B codes for two proteins. Two of the proteins in DNA-A of bipartite begomovirus and in monopartite virus are encoded in the virion sense strand and four in the complementary sense strand. The complementary sense strand proteins are replication-associated protein (REP; AC1), transcription activator protein (TrAP; AC2), replication enhancer protein (REn; AC3), and AC4 protein. Coat protein (CP; AV1) and precoat protein (AV2) are encoded in the virion sense strand. However, the AV2 ORF is absent in new world bipartite begomoviruses [9]. DNA-B contains ORFs BC1 and BV1 encoding movement protein (MP) and nuclear shuttle protein (NSP), respectively. The geminivirus proteins work in coordination to facilitate replication, movement, and anti-defence response to establish a successful infection process [2, 10, 11].

In the infection establishment process, the subviral components of begomoviruses play important roles. Known as alphasatellite, betasatellite, deltasatellites or non-coding satellites, these satellite molecules depend on the helper virus for their replication and propagation, but some of them are adapted in modulating the biological properties of helper viruses [5, 12, 13]. While alphasatellites are self-replicating and depend on the helper virus for encapsidation, movement, and transmission, betasatellites are *trans-*replicated by helper begomovirus and mastrevirus [14–16]. With a genome of nearly 1350 nt, and two proteins βV1 and βC1 encoded in virion and complementary sense strand respectively, the betasatellites take important parts in symptom induction, host defence suppression and insect transmission [17–19]. Besides, betasatellites contain a satellite conserved region (SCR) and an adenine rich region important for betasatellite replication and maintenance [20, 21]. In natural conditions, plants can be infected by multiple viruses, and the stringency of betasatellites associated with their helper virus is very less, which increases the diversity of geminivirus-betasatellite complexes and enhances the probability of disease occurrence in new hosts. Furthermore, the high evolutionary rate of geminiviruses enables them to adapt to new hosts.

Due to the coevolution of plants and the pathogenic viruses, plants acquired multiple strategies to defend and counter viral infection and pathogenesis. However, viruses co-evolve to overcome such resistance responses [10]. Plants control the viruses by preventing the virus gene expression and inhibiting the systemic spread of viruses. Although inhibiting virus gene expression is one of the fundamental ideas of protecting the host from infection, the mainline defence against geminiviruses plays a crucial role in combating the geminivirus infections. This review deals with past and recent findings of major plant immune responses operated against the geminiviruses and a brief discussion on anti-host defence responses. Defence responses such as transcriptional gene silencing (TGS), post-transcriptional gene silencing (PTGS), autophagy, resistance genes and hypersensitive response (HR), protein kinase-mediated immunity, and ubiquitin–proteasome system are discussed in detail. In addition, regulation of phytohormones and alternations in plant primary and secondary metabolism during plant-geminivirus interactions are discussed, and host factors contribute to the pathogen resistance/tolerance are summarised.

## Main text

### Transcriptional gene silencing and RNA polymerase blockers

Plants deploy two major armours against geminiviruses that are based on silencing the expression of viral genes. While the methylation-mediated TGS targets viral minichromosomes, the viral mRNAs are rendered ineffective by PTGS. In the next two sections, we will present the nuances of these two processes.

Geminiviral DNA forms a complex with the coat protein and enters the nucleus. In the nucleus, using the host cell machinery the ssDNAs replicate to the double stranded forms and by binding with host’s histones exist as the minichromosomes [3]. The plant’s response to this invasion is employed by the RNA-directed DNA methylation (RdDM) apparatus to suppress the viral minichromosomes, silencing the viral gene expression by transcriptional gene silencing (TGS) [22, 23]. This epigenetic silencing mechanism involves a sequence of host-virus interactions that reflect the different stages of the infection. In symptomatic tissue, during active replication/expression process, the minichromosomes exist in relaxed conformation, having a chromatin activation marker (H3K4me3) and low level of DNA methylation in comparison to the recovered tissue, where the minichromosomes bear the mark of chromatin-repression (H3K9me2) [24]. The cascade of silencing is operated by a section of small RNAs: siRNAs and miRNAs.

The canonical RdDM pathway is mediated by host DNA dependent RNA pol IV and V, which are evolved from RNA Pol II exclusively to function in plant RNA silencing pathways [25]. RNA Pol IV and V generate 24-nt siRNAs and amplify de novo methylation of target DNA [26]. Pol IV catalyses the formation of single-stranded non-coding transcripts from geminiviral chromatin which are replicated into dsRNA by RNA- DEPENDENT RNA POLYMERASE 2 (RDR2) by CLASSY 1 (CLSY1) dependent manner [26, 27]. These dsRNAs are diced by DICER-LIKE 3 (DCL-3) ribonucleases and generates 24-nt siRNAs duplexes, which are stabilised by HUA- ENHANCER 1 (HEN1), later loaded onto ARGONAUTE 4 (AGO4)/AGO-6 containing RNA-induced silencing complex (RISC). When challenged with beet curly top virus (BCTV), the Pol IV-RdDM machinery reinforced and amplified the viral DNA methylation that was performed by a pathway involving RNA Pol II and RDR6 [26].

RNA polymerase V transcription is independent of siRNA biogenesis and carried out by DDR complex, which includes DEFECTIVE IN RNA DIRECTED DNA METHYLATION 1 (DRD1), DEFECTIVE IN MERISTEM SILENCING 3 (DMS3), REQUIRED FOR DNA METHYLATION 1 (RDM1) and DMS4. The siRNA present in the AGO4-RISC complex base pairs with the transcripts processed by Polymerase V activity. This interaction is stabilized by the AGO4 association with Nuclear RNA polymerase E (NRPE1) carboxyl-terminal tail and KOW DOMAIN-CONTAINING TRANSCRIPTION FACTOR 1 (KTF1) [28]. AGO4 further binds to RDM1 protein of DDR complex and recruits cytosine methyltransferase like DOMAINS REARRANGED METHYL TRANSFERASE 2 (DRDM2) to carry out de novo methylation on the viral genome [29]. Histone modification plays a decisive role in determining the course of host-virus interaction. A histone methyltransferase SU(VAR)3-9 HOMOLOGUE 4 (SUVH4), also known as KRYPTONITE2 establishes the specific repressive epigenetic markers such as histone methylation marks K9, K27 on H3 responsible for transcription repression ultimately results in TGS  [29]. Histone methyltransferase KRYPTONITE (KYP) and DNA methyltransferase CHROMOMETHYLTRANSFERASE 3 maintain the TGS and to overcome this host-mediated TGS, virus-encoded trans-activator AC2 activates an EAR-motif-containing transcription repressor RELATED TO ABI3 and VP1 (RAV2) that represses KYP expression facilitating virus survival in host [30].

The complex transcriptional reprogramming that involves DNA methylation and demethylation is central in the chromatin-based systemic immune responses in plant [31]. The epigenetic studies show the role of RdDM in resistance against geminiviruses [29, 32, 33]. Arabidopsis mutants *ddm1, ago4, drm1drm2, cmt3, adk1* and, *dcl3* that shows reduced viral genome methylation, are hypersusceptible to distinct geminiviruses [32]. However, geminivirus disease complexes overcome the TGS by virus-encoded TGS suppressors. For example, AC2 encoded transactivator protein (TrAP) of bipartite begomoviruses, V2 protein of tomato yellow leaf curl virus (TYLCV), and C2/L2 encoded TrAP of curtoviruses carry out the suppression of TGS pathway [34, 35]. V2 proteins of TYLCV and cotton leaf curl Multan virus (CLCuMuV) directly interact with AGO4 and interfere with binding of AGO4 to the viral DNA, functioning as TGS suppressor and promoter of virulence [36, 37]. Tomato leaf curl Yunnan virus (TLCYnV) encoded C4 protein binds to DOMAINS REARRANGED METHYLASE2 (DRM2) and hampers its binding to viral genome followed by antiviral DNA methylation [38]. Beet severe curly top virus (BSCTV) TrAP protein inhibits the proteasomal degradation of SAMDC1 (S-adenosyl-methionine decarboxylase). This disturbs the ratio of SAM (S-Adenosyl-methionine)/dSAM (decarboxylated SAM), which leads to inhibition of geminiviral DNA methylation [39]. Furthermore, TrAP protein inhibits ADK that is involved in the production of SAM, a methyl donor [34]. TrAP and βC1 protein interact with SAHH (S-adenosyl homocysteine hydrolase), which is responsible for maintaining the methyl cycle during TGS [40] and dampens TGS. To increase the susceptibility, TYLCV pre-coat protein competes with Methytransferase 1 (MET1) and interacts with HISTONE DEACETYLASE 6 (HDA6) to repress DNA methylation [41]. On the plants’ front, a total control of transposon elements and compaction of chromatin are achieved by Pol IV-RdDM mediated TGS of viral genome involving Pol IV and Pol V [42].

Post-translational modification of histone, an inherent gene expression regulatory process of plant is used by *Arabidopsis* against viral pathogens. EMSY-LIKE 1 (EML1) is a histone reader protein binds to H3K36 modification sites on viral chromatin blocking the access of RNA pol-II to the viral genes and suppressing the expression [42]. The access of RNA pol-II to viral genes is inhibited by *Solanum lycopersicum* regulatory particle triple-a atpase 4A (RPT4a), a subunit of 26-proteasome protein, that binds to the intergenic region of tomato leaf curl New Delhi virus (ToLCNDV), inhibiting the viral transcription [43].

All these molecular dynamics are inspiring developing newer strategies against geminivirus infections. Stable or transient expression of invert repeat constructs to the homologous sequence of geminivirus promoter region inhibits the expression of downstream genes and leads to the reduced viral load as well as symptom recovery. IR region/bidirectional promoter region has been successfully employed in generating the target-specific siRNA to downregulate the virus gene expression [44]. RNA-dependent RNA polymerase 1 (RDR1) of *Nicotiana tabacum* enhances cytosine methylation of tomato leaf curl Gujarat virus (ToLCGV) promoter and represses the virus gene expression and increases virus specific siRNA accumulation eventually leads to symptom remission [45]. Moreover, *NtRDR1* overexpression in *N. benthamiana* alters the expression of host defence genes such as subunit-7 of COP9 Signalosome (CSN) complex, WRKY6 and USPA-like protein and confers reduced susceptibility to ToLCGV infection [46]. Administration of bidirectional promoter fragment from DNA-A of vigna mungo yellow mosaic virus (VMYMV) into VMYMV infected *V. mungo* plants abolished viral DNA accumulation and lead to disease recovery [47].

### Post transcriptional gene silencing

The RNA transcripts produced by the viruses are targeted by the cytoplasmic siRNA-mediated silencing pathway of plant. This post-transcriptional gene silencing (PTGS) is a sequence-specific mechanism, is crucial for the host gene expression, development and defence [48]. In response to viral transcripts inside the cells, the PTGS initiates to target the dsRNA segments derived from either complementary viral transcripts (usually the products of bidirectional transcription) or viral RNA secondary structures like hairpins. DICER- LIKE protein (DCL) and dsRNA binding protein (DRB) recognise and process the dsRNAs into 21–24-nt siRNAs. HUA ENHANCER 1 (HEN1) protein methylates 3′ end of siRNAs and protects them from 3′ to 5′ exonucleolytic degradation and uridylation [49]. Alongside, these small RNAs duplexes are recruited onto AGO proteins to provide sequence specificity for targeting and forms RISC complex, resulting into mRNA degradation by cytoplasmic exonucleases or translation inhibition [50]. A second wave of amplified PTGS is generated at the systemic sites by the primary siRNAs to induce systemic resistance [25]. To counter this robust immunity response, geminiviruses have co-evolved several suppressors which interfere at multiple stages of the siRNA pathways such as sensing and activation of PTGS, siRNA biogenesis, amplification and systemic spread to mitigate the host defence [51]. Nuclear shuttle protein (NSP), encoded by the ORF BV1, induces ASYMMETRIC LEAVES2 (AS2) expression in the infected cells that enhances the decapping activity of DECAPPING 2 (DCP2), accelerating the mRNA turnover and hindering siRNA accumulation as well as host RNA silencing [52]. An endogenous RNAi suppressor calmodulin-like protein (CaM) is upregulated by βC1 protein, triggering an interaction cascade that leads to degradation of Suppressor of Gene Silencing 3 (SGS3) and suppression of RDR6 activity, eventually affecting the anti-viral RNA silencing process [53, 54]. Rep protein of wheat dwarf virus (WDV) binds to 21nt and 24nt siRNAs duplexes, inhibiting local and systemic silencing of viral RNA and spread of signals [55]. TYLCV infected and cotton leaf curl Multan betasatellite (CLCuMuB) βC1 expressing transgenic plants showing increased expression of AGO1 and DCL1 underscore the nuanced anti-PTGS process in play [56]. CLCuMuV C4 interacts with the core enzyme of methyl cycle, S-adenosyl methionine synthetase (SAMS) to inhibit TGS and PTGS and, C4^R13A^ mutant fails to retain the suppressor activities [57]. SAMS utilises ATP for converting the methione to SAM [58]. Intriguingly, the arginine 13 of cotton leaf curl Kokhran Virus-Dabawali (CLCuKV-Dab) C4 protein had shown to be important for ATPase function [59]. Presumably CLCuMuV C4 exerts its ATPase action to inhibit the SAMS activity.

Exploitation of host PTGS constitutes a promising strategy in rising the potential defence strategies against geminivirus. In fact, this strategy successfully introduced three decades ago, for developing resistance against a plant RNA virus, tobacco mosaic virus (TMV), where the transgenic expression of TMV CP protein delayed the disease progression [60]. Similar result was also observed with TYLCV. However, the protection was dependent on the expression of the transgene in infected CP transgenic tomato plants [61]. Recombinant vector-mediated expression of artificial dsRNAs raised from either conserved or fusion transcripts belong to same or different virus origin triggers siRNA accumulation and potentially triggers PTGS against broad spectrum geminiviruses [62, 63].

### MicroRNAs in antiviral immunity

MicroRNAs (miRNAs) play a significant regulatory role in plant development as well as  biotic and abiotic stress responses. In plants, miRNA biogenesis predominantly occurs in the following steps: (1) Transcription of primary miRNA from *MIRNA* *(MIR)* genes by RNA polymerase II, (2) Processing of primary miRNAs to nascent miRNA by Dicer-like proteins, (3) Methylation of nascent miRNA and assemble into RISC and, (4) Binding to target mRNA and regulation of gene expression [64]. Existing literature highlights the role of miRNAs against geminiviruses as an underexplored area with promising insights on several aspects of plant-virus interaction. Transient or transgenic expression of geminiviral proteins often exhibit phenotypic abnormalities, evidences the possible involvement of perturbations in miRNA regulatory pathways [65, 66]. AC4 protein of african cassava mosaic virus (ACMV) directly binds to the matured miRNAs and interferes with the mRNA homeostasis that results into developmental abnormalities [66]. The reports on tomato yellow leaf curl Sardinia virus (TYLCSV) and mungbean yellow mosaic India virus (MYMIV) infection in tomato and mungbean, respectively highlighted the host miRNAs that targets phytohormone pathways, resistance (R) genes, receptor-like serine/threonine-protein kinases and transcriptions factors involved in the development [67, 68]. The influence of betasatellite on induction of host miRNA has been studied in the plants co-infected with tomato yellow leaf curl China virus (TYLCCNV) and tomato yellow leaf curl China betasatellite (TYLCCNB), in the presence and absence of functional *βC1*. TYLCCNB responsive miRNAs such as miR391, miR397, and miR398 have been predicted to generate the phased secondary siRNAs (phasiRNAs) [69]. Bioinformatics analysis suggested the tendency of host miRNAs to bind to the geminiviral genome and ORFs and may negatively regulate viral transcription [70]. Prediction analysis of RNA hybrid software revealed miR/miR* sequences are capable of binding ToLCNDV ORFs includes AC1, AC2, AC3, AV1, AV2, BV1 and to betasatellite non-coding region at one or more than one site. Nonetheless, still virus dominates the host defence response by successfully deploying its silencing suppressors [70]. Transgenic plants expressing miRNAs specific to AV1 and AV2 proteins confer tolerance to tomato leaf curl virus (ToLCV), indicating the effectiveness of miRNAs against geminiviruses [71]. *Gossypium hirsutum* miR398 and miR2950 were found to bind to the genomes of both CLCuMuV and CLCuMB, and potentially augmented the CLCuD resistance in transgenic plants [72]. In silico analysis suggests the binding capability of *Glycine max* miRNAs on the genome of MYMIV and mungbean yellow mosaic virus (MYMV) but also involves in regulation of plant defence responses [73]. A stable barley transgenic line, developed with a polycistronic artificial miRNA, gains the resistance against WDV at a lower temperature ranging between 12–15 °C [74]. Recently, ToLCV resistant tomato transgenic lines have been generated by overexpressing the ATP binding domain of AC1 protein via artificial miRNA without compromising the yield [75]. Expression analysis of miRNAs sheds light on possible role of Argonaute homeostasis along with miRNA directed cleavage of virus movement protein in developing resistance against viruses along with the gene regulatory changes in hormonal signalling pathways [76]. Greater supplementary research is required to understand miRNAs as a potential tool in rising defence against geminiviruses.

### Ubiquitin-proteasomal pathway and SUMOylation

Ubiquitination is a post-translational modification process, where the protein ubiquitin is conjugated to the lysine moiety of a target protein and eventually directs the protein to 26S proteasomal degradation. Ubiquitination requires the sequential action of three enzymes- ubiquitin-activating enzyme (E1), ubiquitin-conjugating enzyme (E2), and E3 ubiquitin ligase (E3). One of the most abundant E3 ligase families comprises Cullin Ring Ligases (CRLs) in SCF complex (SKP1-CUL1-F-box-protein) which is regulated by CSN complex. The F-box proteins contribute to hormonal regulations of plants [77]. The F-box protein CORONATIN INSENSITIVE 1 (COI1) (SCF^COI1^), functions as one of the components of the jasmonic acid (JA) receptor, is involved in pathogenesis in plants. Upon pathogen infection, increased accumulation of jasmonoyl isoleucine (JA-Ile) facilitates the interaction of repressor protein JAZ (Jasmonate Zim domain) with SCF^COI1^ which cause degradation of JAZ proteins, and elevated the expression of JA responsive genes [78] that were earlier repressed by JAZ.

**Fig. 1:**
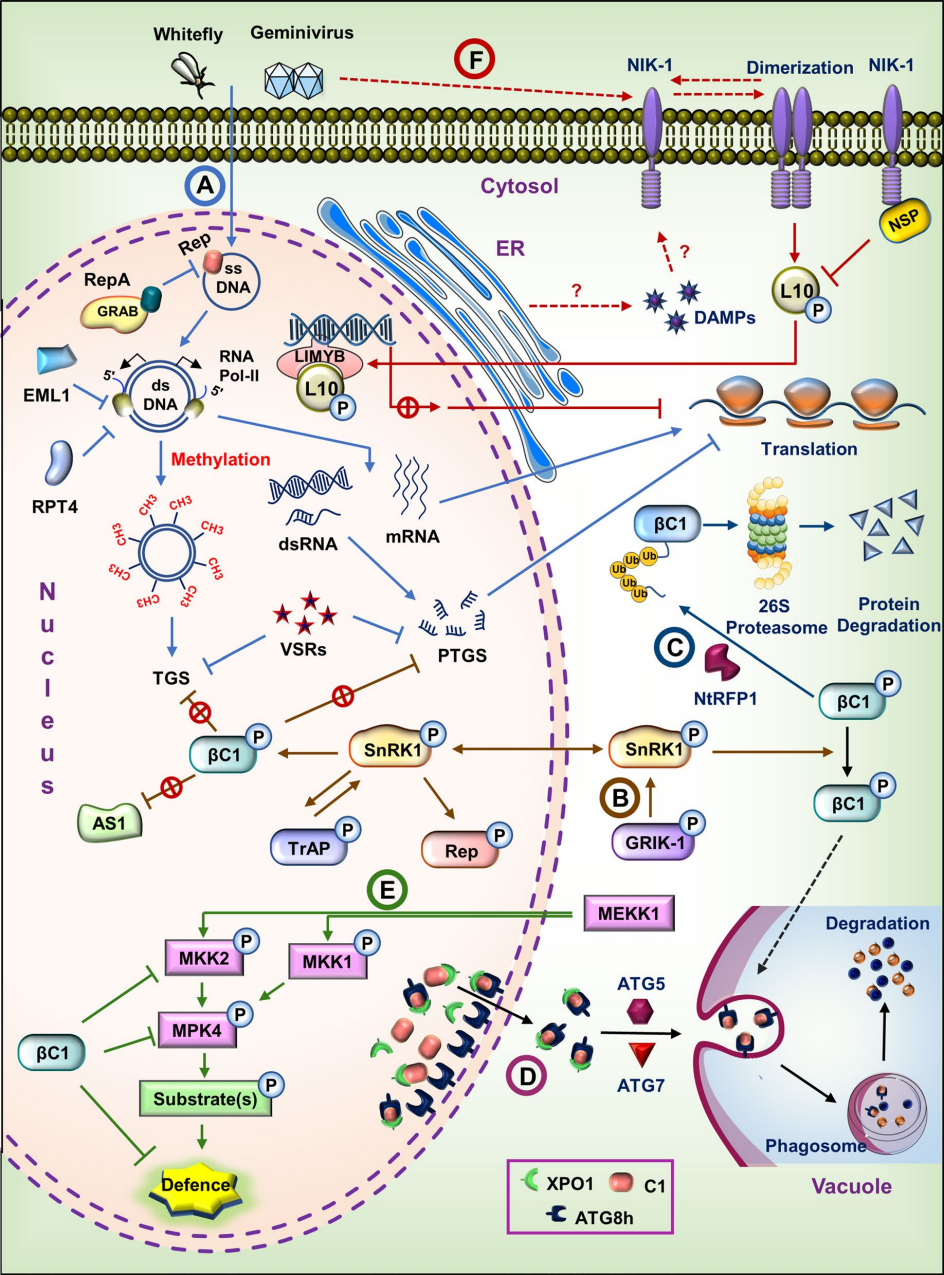
Schematic overview of plant immune strategies against geminiviruses. Geminivirus infection initiates with the release of viral ssDNA into the nucleus, subsequently leads to the replication, transcription and translation of viral genome. (**A**) Plants counteract geminivirus genetic life cycle via multiple host factors. GRAB interacts with RepA and interferes with the replication. RPT4a and EML1 hamper the geminivirus active transcription by obstructing the RNA Pol-II on virus euchromatin. Additionally, host induces RNAi via TGS and PTGS to suppress the viral gene expression. Virus-encoded VSRs potentially suppresses the RNAi. (**B**) Geminivirus induced GRIK1 autophosphorylates and activates SnRK1 which interact and phosphorylates the viral Rep, TrAP (AL2/C2) and βC1 protein. Phosphorylation of Rep and TrAP impedes Rep binding and causes a delay in the infection, respectively. βC1 phosphorylation hampers the TGS and PTGS suppressor functionalities and attenuates symptom expression via suppression of AS1-βC1 mediated downstream responses. Phosphorylated βC1 may also direct to autophagy. (**C**) Tobacco RFP1 interacts with βC1 and prompts the βC1 degradation via ubiquitin-mediated 26S proteasomal pathway and causes the symptom attenuation. (**D**) ATG8h interacts with nuclear C1 and translocate to cytosol XpoI dependent manner. The ATG8h-C1 complex is then recruited into autophagosomes with the aid of ATG5 and ATG7 for vacuolar degradation. (**E**) Defence regulated MEKK1-MKK1/MKK2-MPK4 module induced, activated by geminivirus infection and exerts the basal defence response. However, βC1 protein directly interacts with MKK2 and MPK4, thereby suppress the broad spectrum of downstream defence reactions. (**F**) NIK-1 from plasma membrane activated upon the geminivirus infection triggers dimerization and autophosphorylation. Alternatively, PTI induced DAMPs secreted from ER in response to virus attack may cause NIK-1 activation. Active NIK-1 phosphorylates and translocate L10 into the nucleus where it binds to LIMYB to block the transcription of ribosomal biosynthesis genes which affects the global translation and prevents the translation of viral genes

In plants, the quality control process of protein involving proteases, autophagy and proteasomal degradation systems work closely with defence pathway that requires degrading the pathogenic proteins [79, 80]. During geminivirus infection, aggregation of viral proteins in the cytosol and nucleus [81, 82] is reported often. These aggregates sequester the viral proteins and virion particles from the host immune sensors to ensure the survival, multiplication and movement of the viruses [83]. A number of reports suggest that Ubiquitin-proteasomal pathway regulates geminiviral infection by degrading either viral or cellular proteins [79, 84, 85]. NtRFP1, a tobacco RING -finger protein, which functions as a ubiquitin E3 ligase interacts with βC1 protein and mediates βC1 ubiquitination, attenuating betasatellite mediated symptom expression [79] (Fig. 1). Ubiquitin activating enzyme (UBA1) interacts with TrAP protein and silencing of UBA1 promotes early viral infection in transgenic *N. benthamiana* [85]. TYLCV TrAP protein also regulates CSN activity to inhibit SCF^COI1^ [86]. βC1 protein interrupts SKP1 and CUL1 interaction during CLCuMuV infection disrupting the proteasomal degradation pathway and altering plant hormonal signalling cascades [87]. *S. lycopersicum* E2 enzyme UBC3 (Ubiquitin-conjugating enzyme 3) activity is also blocked by βC1, with aftermath of decreased level of total polyubiquitinylated protein and increased symptom severity [88]. Cyclin-dependent kinase/cyclins control cell cycle progression in plants and animals. CDK inhibitors (CKIs) negatively regulate CDK/Cyclins. One of the mammalian CKIs, CKI p27*kip1*, is degraded through the help of ubiquitin ligase KPC (Kip-1 ubiquitination promoting complex). Expression of C4 gene of BSCTV in *Arabidopsis* induces Ring finger protein RKP1 the protein similar to human KPC1. RKP1 acts as an ubiquitin E3 ligase and interacts with CKIs, thus lowering the protein level of CKIs during the infection with the effect of continued cell cycle progression [84]. BSCTV also couples ubiquitin-proteasomal system (UPS) to TGS defence pathways, hampering the latter [39]. Numerous molecular studies also revealed the involvement of UPS in regulating the immune responses by altering the fate of transcriptional regulators [77], virus replication [89] and, movement [90]. However, these roles need to be ascertained in the context of geminivirus infection.

SUMOylation is a transient, post-translational modification, similar to ubiquitination and is involved in ligation of a 10 kDa small ubiquitin-related modifier, SUMO to the lysine residues of target peptides to modulate protein activities and interaction as well as subcellular localization [91]. The dynamic equilibration of SUMOylation plays a crucial role in development [92], biotic and abiotic stress responses [93] of plants. Interactions of SUMO conjugating Enzyme 1A (NbSCE1), E2-SUMO conjugating enzyme of *N. benthamiana,* with N-terminal of Rep proteins of tomato golden mosaic virus*,* (TGMV), TYLCSV*,* and ACMV are vital for virus replication [94, 95]. In plant RNA virus infection, interaction of SCE1 and viral replicases has a similar positive-effects on virus replication [96]. The interaction between NbSCE1 and Rep/AL1 protein in both monopartite and bipartite begomoviruses augments virus replication, probably by altering the SUMOylation patterns of specific host factors to create a favourable environment for the viruses [95]. SUMOlyation of proliferating cell nuclear antigen (PCNA), the replication processivity factor is compromised by the Rep protein creates a similar permissive ambience for geminivirus replication [97]. Synedrella yellow vein clearing virus (SyYVCV)- βC1 undergoes via ubiquitination mediated degradation [91]. The N-terminal SUMOylation motifs of βC1 functions as stability markers whereas the C-terminal SUMO interacting motifs (SIMs) binds to the host cellular components, promoting the protein degradation. To counter the host-mediated degradation, βC1-protein interacts with *Nb*SUMO1 and recruits the host SUMOylation machinery. Both N-terminal and C-terminal SUMOylation motifs of βC1 are indispensable for the symptom expression, virus replication and systemic movement. However, chloroplast localization of βC1 solely depends on C-terminal SUMOylation motifs [91]. Further research is required to explore how the plants engage the defence response to counteract such virus induced microenvironmental modifications.

### Autophagy as a viral venator

Autophagy is an evolutionary conserved process of recycling the degraded or undesirable cellular components taking place in the cell. Autophagic cargo sequestered into vesicle-like compartments and subsequently fused with lytic components such as lysosomes in animals and vesicles in the plant cells. Studies conducted on plant DNA and RNA viruses confirm that autophagy plays a potential antiviral role in host innate and adaptive immunity [98, 99]. In vivo and in vitro experiments showed that CLCuMuB βC1 protein interacts with autophagy-related protein NbATG8 through its ATG8 interacting motif (LVSTKSPSLIK) and directs it for degradation. Disruption of βC1-ATG8 interaction by a point mutation (V32A) in the ATG8 interaction motif promotes the virus replication and disease symptoms. Since ATG genes are functionally redundant, silenced ATG5 and ATG7 transgenic plants when infected with CLCuMuV and associated betasatellite showed severe and early symptoms [98]. Furthermore, interaction of ATG8h protein with Rep protein of TLCYnV leads to ATG8h mediated Rep translocation to cytoplasm and degradation [80] (Fig. 1).

Autophagy carries out both pro-viral and anti-viral roles in host cells to maintain the balance of cellular and viral proteomes. Geminiviruses have recently been reported to manipulate autophagy-mediated defence by inducing autophagy of host factors involved in other defence pathways. TYLCCNB-encoded βC1 regulates Nbrgs-CaM, which induces degradation of NbSGS3 with the help of ATG factors [54]. βC1 protein of CLCuMuB disrupts the interaction between a negative autophagic regulator and ATG3 protein to induce autophagy in *Nicotiana benthamiana* [100]. Further research is needed to reveal the mechanism behind the regulation of autophagy during viral infection and explore the potential of blocking proviral autophagic pathways as a mean to control the infection.

### Kinases as transducers of defence

Protein kinases are some of the key components involved in plant growth, development and defence including pathogen sensing and defence response induction [101]. Protein kinases are accountable for setting different signalling cascades in motion for efficient plant defence against geminiviral infection [102–104]. Viral proteins modulate the signal transduction pathways via both direct and indirect interactions with different host protein kinases. This section illustrates various mechanisms by which protein kinases such as SnRK1, MAP kinases and receptor like kinases (RLKs) orchestrate the cellular responses during plant-virus interactions.

### SnRK1 mediated signalling

The SUCROSE NON-FERMENTING1-related protein kinase 1 (SnRK1) is a Ser/Thr kinase that functions as an energy sensor and central regulator of energy, metabolism and stress responses. It operates multi organellar crosstalk and potentially regulates downstream transcription factors involved in diverse molecular pathways to maintain cellular homeostasis [105]. Upon cabbage leaf curl virus (CaLCuV) and BCTV infection, functionally redundant geminivirus Rep interacting kinases GRIK1 and GRIK2 expression gets enhanced, which causes SnRK1 phosphorylation and activation [106]. GRIK1 and GRIK2 also interact with Rep protein of TGMV [107]. The lower expression of SnRK1 enhances the susceptibility of plants towards geminivirus, whereas SnRK1 overexpression leads to an increased resistance in plants [103]. SnRK1 phosphorylates TYLCCNB encoded βC1 protein at serine 33 and threonine 78 residues, which negatively impacts the titres of both helper virus and betasatellite as well as disease development in *N. benthamiana* [108]. The phosphorylation of βC1 also suppresses its role in suppression of methylation mediated RNA silencing, which further explains the significant role of SnRK1 against geminiviruses (Fig. 1). SnRK1 may also induce autophagy of βC1 protein as yeast, and mammalian homologs of SnRK1 have been reported to promote autophagy by phosphorylation of different protein substrates [98, 109]. AtREM4 (*Arabidopsis thaliana* remorin group 4), which functions as a positive regulator of the cell cycle during BCTV and BSCTV infections, gets phosphorylated by SnRK1 and induce the degradation of AtREM4 by 26S proteasomal degradation pathway [110].

Geminiviral TrAP proteins AL2 from TGMV and L2 from BCTV interact with *Arabidopsis* SnRK1 and inhibit its activity to enhance pathogenesis [111]. SnRK1 maintains balance of cellular metabolic energy of host cells to defend against viral infection. Therefore, depletion of ATP or increased level of 5′-AMP activates SnRK1. As Adenosine kinase (ADK) phosphorylates adenosine to 5′-AMP, L2 and AL2 interact with ADK and disable the SNF1 kinase-related antiviral mechanism [111]. TrAP protein of CaLCuV also gets phosphorylated by SnRK1 at serine 109 position, which leads to delayed viral infection [33]. Further, SnRK1 phosphorylates TGMV-Rep at serine 97 position hindering binding of Rep onto the viral genome and inhibiting viral infection [112]. As suggested by the recent reports, being a global energy regulator of the cell and carrying out the role of metabolic modulator, SnRK1 has emerged as a pivotal player in plants antiviral defence armour.

### MAP kinase cascade

Mitogen-activated protein kinase (MAPKs) are widely studied, and known to be involved in signal transduction and signal amplification processes and defence against diverse phytopathogens as well as in abiotic stresses [113]. MAPKs are activated by MAPK Kinase, which gets regulated through cross-phosphorylation. The role of MAPK cascades in plant innate immunity against bacterial and fungal pathogens is well studied [114]. New findings are highlighting the role of MPKs in antiviral defence responses, too*. Vigna mungo* MAPK1 has been found to suppress MYMIV accumulation and upregulate salicylic acid (SA) mediated expression of pathogenesis-related (PR) genes [115]. Similarly, MAPK3 silenced tomato plants showed reduced tolerance to viral infection and attenuated expression of SA/JA regulated defence related genes [116]. Earlier, global transcriptional analysis of whitefly after TYLCCNV infection revealed the downregulation of genes involved in MAPK signalling pathways [117]. TYLCCNV infection leads to activation of MAPK signalling cascade for defence, but βC1 protein interacts with MKK2 and MPK4 inhibiting the kinase activity and limiting the anti-viral activity of MAPK [104] (Fig. 1). Recently, C4 mediated suppression of MAPK cascade activation has been discovered in *N. benthamiana *[118]. TLCYnV-encoded C4 competes with BRASSINOSTEROID INSENSITIVE 1 (BRI) to bind BRI1 KINASE INHIBITOR1 (BKI1) and stabilizes the protein complex on plasma membrane. The unavailability of free BIK1 precludes the autophosphorylation of ERECTA (ER), concomitantly leading to the inhibition of downstream MAPK cascade activation which facilitates optimal conditions for TLCYnV infection [118].

### Receptor-like kinases

RLKs are transmembrane proteins that transduce extracellular signals by their specific ligand binding domains, a membrane-spanning region, and cytoplasmic serine-threonine kinase domain to regulate cell differentiation, patterning, development and innate immunity [119]. They act as pattern recognition receptors (PRRs) recognizing microbe-associated molecular patterns (MAMPs) and initiating the basal innate defence responses [119]. RLKs also recognize secondary danger signals produced in a stressed situation in the cells that boost the immune response against pathogens [120]. One of the RLKs, NSP interacting kinase (NIK) is encoded by a small multigenic family that consists of three genes NIK1, NIK2, and NIK3. Geminiviral nuclear shuttle protein (NSP) acts as a target of NIKs, implicating the existence of RLKs mediated immune response against geminivirus [121]. The plants deficient with *nik* exhibited enhanced susceptibility to begomoviruses infection [122]. Geminivirus infection triggers NIKs oligomerization and transphosphorylation of kinase domain at T474 that activates NIK1 kinase prompting the latter to phosphorylate the cytoplasmic ribosomal protein 10 (RPL10) [102]. As the phosphorylated RPL10 translocates into the nucleus with the transcription factor LIMYB (L10-interacting MYB domain-containing protein), it forms RPL10-LIMYB complex that negatively regulate the virus infection by binding to the promoters of ribosomal protein gene and represses the expression (Fig. 1). However, the host NIKs mediated resistance against geminiviruses is limited by the NSP of geminiviruses as it interacts with NIKs and suppresses its activity [122] (Table 1). Another RLK, CLAVATA 1 (CLV1) that regulates WUSCHEL gene expression and helps in maintaining the meristem, undergoes binding by the S- acylated form of BSCTV C4 protein; an interaction leading to anomalous siliques development in *Arabidopsis* [123]. As it localizes to the plasmodesmata as well as to the plasma membrane, BARELY ANY MERISTEM 1 (BAM1) and BAM2 helps in expanding systemic movement of RNAi signals and thus obstructing the spread of the virus to other cells. However, TYLCV C4 protein binds to BAM1 and inhibits the propagation of silencing signals [124]. *Arabidopsis* Shaggy like kinase protein AtSK21, also known as AtBIN2 (Brassinoids inhibitor 2), negatively regulates brassinosteroid signalling and affects male sterility [125]. During sweet potato leaf curl virus (SPLCV) infection, viral C4 protein targets AtBIN2 inducing anomalous development including male sterility in *Arabidopsis* [125]. C4 physically interacts with RLKs, FLAGELLIN SENSING 2 (FLS2) and BRI1 and affect the downstream pathways as the interaction reduces the time of apoplastic ROS burst without influencing downstream marker genes expression [126]. Shaggy-related protein kinase (SKη) also determines C4 mediated symptom induction. The affinity of the NbSKη-C4 interaction and tethering to the plasma membrane complex regulates the viral pathogenicity [127]. RLK Proline-rich extension-like receptor kinase (PERK) like protein is exploited by the viral machinery to positively regulate viral protein NSP and enhancing the infection of tomato crinkle leaf yellows virus (TCrLYV) and TGMV. PERK can be considered as a potential resource to develop viral resistance in plants as T-DNA insertional mutation in PERK attenuates infection [128]. Various other RLKs like PHLOEM INTERCALATED WITH XYLEM members (PXYs), PEP1 RECEPTOR members (PEPRs) are some potential targets of viral C4 proteins. Manipulation of such RLKs which mediate defence and developmental processes by geminivirus points towards the possible roles of these RLKs in antiviral activity [129]. RLKs, being critically important in the perception of pathogens, need a broader exploration to reveal the molecular and genetic pathway against geminiviruses.

**Table 1:** Host factors involved in the antiviral defence against geminiviruses

Host	Host factor	Function	Viral factor	Geminivirus	Precise role in defence response	References
*Host defence responses*						
*A. thaliana*	SnRK1	It is a global regulator of energy metabolism during growth and stress conditions	Rep	TGMV	SnRK1 phosphorylates Ser 97 of Rep protein and interferes replication and infection	[98]
			TrAP	CaLCuV	SnRK1 phosphorylates AL2 at ser 109 that delays infection process	[33]
*N. benthamiana*	PsbP	It is a core protein of oxygen-evolving complex that stabilizes Photosystem II	βC1	ToLCNDV/RaLCB	PsbP binds to the viral genome and reduces virus replication	[191]
*N. benthamiana*	ATG8	It is involved in autophagy, mediates protein degradation	βC1	CLCuMuV/CLCuMuB	ATG8 binds to βC1 and degrades via autophagy thus causes reduction in viral titre	[84]
*S. lycopersicum*	ATG8h	It is an autophagy factor, mediates protein degradation	Rep	TLCYnV	C1 gets exported to the cytoplasm by ATG8h and degraded which restricts viral infection	[66]
*N. tabacum*	RFP1	It is an E3 ligase that mediates protein ubiquitination	βC1	TYLCCNV/TYLCCNB	RFP1 interaction with βC1 leads to βC1 degradation thus reduces viral infection	[65]
*S. lycopersicum*	UBA1	A ubiquitin-activating enzyme, catalyses the first step in 26S proteasomal degradation pathway	TrAP	TYLCSV	Silencing of UBA1 increases viral infection. TrAP protein interacts with UBA1, thus inhibit its defensive activity	[71]
*A. thaliana*	EML1	A histone reader protein, represses active transcription by binding on H3K36 modification	Viral minichromosome	CaLCuV	EML1 binds to viral minichromosome and blocks transcription, therefore attenuates infection	[39]
*S. lycopersicum*	RPT4a	A subunit of 26S proteasome, aid in unfolding target proteins	Intergenic region	ToLCNDV	RPT4a binds to the viral intergenic region and blocks transcription	[40]
*V. mungo*	MAPK1	A protein kinase involved in SA mediated defence	Not known	MYMIV	It mediates defence by inducing SA responsive genes	[101]
*Triticum monococcum*	GRAB	A Geminivirus Rep A-Binding protein. It may have role in plant development	RepA	WDV	GRAB1 and GRAB2 bind to C-terminus of RepA and inhibit replication	[193]
*N. benthamiana*	*Nb*SUMO1	A component of SUMOylation system	βC1	SyYVCV/ SyYVCB	It promotes ubiquitin mediated degradation of βC1 protein	[77]

Host	Host factor	Function	Viral factor	Geminivirus	Remarks	References
*Geminivirus adaptations to host defence*						
*N. benthamiana*	HDA6	It interacts with MET1 and maintains chromatin silencing	Pre-coat protein	TYLCV	Pre-coat protein interfere with MET1 interaction to HDA6 and inhibit TGS	[38]
*A. thaliana*	ADK	It converts adenosine to AMP which is essential to maintain the methyl cycle during TGS and aid in the activation of SnRK1	TrAP	TGMV, BCTV	TrAP inactivates ADK and causes TGS suppression and inactivation of SnRK1 defence pathway	[34]
*A. thaliana*	SAHH	It catalyses the generation of methyl donor during TGS mediated methylation of viral genome	βC1	TYLCCNV/TYLCCNB	βC1 inhibits SAHH enzyme and suppress TGS	[37]
*A. thaliana*	BAM1	It facilitates cell to cell movement of RNAi signals	C4	TYLCV	C4 interacts with BAM1 domain and inhibits spread of silencing signals	[109]
*N. benthamiana*	MKK2 MPK4	Regulates defence responses by via phytohormones, ROS and defence-related gene expression during biotic stress	βC1	TYLCCNV/TYLCCNB	βC1 inhibits the kinase activity of both MKK2 and MPK4 and increases host susceptibility	[90]
*A. thaliana*	SnRK1	It is a global regulator of energy metabolism during growth and stress conditions	TrAP	TGMV, CaLCuV	TrAP inactivates SnRK1 and leads to enhanced susceptibility	[97]
				BCTV		[98]
*A. thaliana*	NIKs	It undergoes reversible phosphorylation within its activation loop domain to carry out the defensive function	NSP	CaLCuV	NSP inhibits autophosphorylation of NIK and thus breaks suppression of NIK mediated global protein synthesis	[88]
*A. thaliana*	CSN5	It regulates SCF complex activity during ubiquitination	TrAP	TYLCV, TYLCSV	TrAP interacts with CSN5 and impairs SCF complex function thereby inhibits jasmonic acid signalling and enhanced infection	[72]
*N. benthamiana*	*Nb*SUMO1	A component of SUMOylation system	βC1	SyYVCV/SyYVCB	It stabilises the βC1 protein	[77]

### Phytohormone modulations

Phytohormones not only regulate various physiological activities related to development, metabolism, reproduction but they are also essential in management of abiotic and biotic stresses [130, 131]. Various phytohormones like salicylic acid (SA), jasmonic acid (JA), and ethylene have been known to work either synergistically or antagonistically to generate diverse host defence responses against pathogens. Crucial roles of JA, SA against geminiviruses have been elucidated and the involvement of auxin, cytokinin, gibberellic acid, brassinosteroids and abscisic acid in the anti-virus activity are being explored [132].

SA is synthesized in plants during biotic stress and establishes both local and systemic acquired resistance (SAR) via synthesis  of PR proteins. CaLCuV infected *A. thaliana* transcriptome analysis revealed activation of the SA pathway during infection as *Arabidopsis cpr1* plants that exhibited high endogenous SA level and increased PR proteins expression were less susceptible to CaLCuV infection [133]. Similarly, overexpression of GLUTAMINE DUMPER 3 (LSB1/GDU3), a gene important in amino acid transport, activates the SA pathway and weakens DNA replication of BSCTV [134]. However, during TYLCSV infection in *S. lycopersicum*, biosynthesis of SA has been reported to be reduced. SA minimises the egg hatchability of vector whitefly putting pressure on viral propagation [135]. Recent report suggests induction of expression of SA responsive PR genes (*SlPR1, SlPR2, and SlPR5*) and ROS scavenging enzymes (*SlSOD, SlPOD, SlCAT2*) following TYLCV infection in *S. lycopersicum* which contributes to increased resistance against the virus [136].

Methyl jasmonate (MeJA) treated plants also developed milder symptoms and low viral titre compared to the control plants when infected with BCTV [86]. Tomato plants infected with TYLCSV had several JA responsive genes upregulated including JA signaling pathway gene *COI1* but had the lower level of *JASMONATE INSENSITIVE1* (JAI1), a transcription factor activated by the JA [67]. *N. tabacum* transgenic plants expressing TrAP protein had increased expression of JA biosynthetic genes as well. Although betasatellite encoded βC1 protein did not have a direct impact on JA biosynthetic genes, it represses JA downstream marker genes such as *PLANT DEFENSIN1.2* (*PDF1.2*), *PATHOGENESIS* *RELATED4* (*PR4)* and *CORONATINE INSENSITIVE13* (*CORI3*)*,* thus hampering the hormonal defence suppression mechanisms [137]. As βC1 interacts with MYC2, the MYC2-mediated JA responses gets suppressed [138]. Activation of JA leads to reduced development of whitefly *B. tabaci* nymphaea; however, the adult population can suppress JA related defence [139].

Gibberellic acid (GA) signalling is mediated via the degradation of DELLA proteins, which are negative growth regulators. GA biosynthesis and GA receptors genes were found to be upregulated in tomato plants infected with TYLCSV while the repressor protein of GA, Gibberellic-Acid Insensitive (GAI) downregulates, suggesting the fine tuning of GA homeostasis during the geminiviral infection [67]. CLCuMuB βC1, when interacts with the DELLA protein, represses its degradation, affecting the GA response pathway.

Role of classic growth hormones like auxins and cytokinins against geminiviruses are yet to be explored conclusively. Auxin is a pivotal regulator of growth and development of stem and roots as responsiveness to light and temperature. Through its precursor Tryptophan, auxin signalling is related to chemical defence pathways involving camalexins and glucosinolates that selectively inhibits the growth of necrotrophic and biotrophic pathogens [140]. ToLCNDV-encoded AC4 disrupts endogenous auxin content and downstream signaling cascade in tomato by interacting with auxin biosynthetic enzymes such as TAR1 (tryptophan amino transferase 1)-like protein, CYP450 monooxygenase. It also upregulates the expressions of miRNAs that target auxin signaling components, reprogramming the virus replication and altering the symptom manifestation [141]. RNA sequence analysis of WDV infected samples showed downregulation of auxin-induced protein 15A and auxin-responsive protein SAUR72 compared to the control plants. Small auxin upregulated RNA (SAUR) family proteins are involved in the Auxin/Indole-3-acetic acid (AUX) signalling pathway. The downregulation of SAUR72 suggested the possible roles of auxins in antiviral response. Similarly, two-component response regulator encoding ORR22 and ORR4 genes that are involved in cytokinin signalling pathways were upregulated and downregulated, respectively [142]. TGMV TrAP protein and spinach curly top virus (SCTV) C2-encoded TrAP protein expression resulted in inhibition of activity of ADK, a regulator of primary cytokinin responsive genes. Inhibition of ADK prevents cytokinin nucleosides phosphorylation leading to the accumulation of more bioactive cytokinins [143]. This increases the cell division rate and promotes severe infection. Regulation of level of cytokinins may decisively control the susceptibility of plants towards geminiviruses.

Abscisic acid is a widely studied phytohormone in plant abiotic stress tolerance. However, the correlation between geminivirus infection and abscisic acid has largely remained unexplored. Exogenous application of abscisic acid and auxin induces expression of *A. thaliana* homeobox ATHB7 and ATHB12 genes that encode homeodomain-leucine zipper family transcription factors. Similar genes were found to be induced during BSCTV infection [67]. Since expression of ATHB12 in BSCTV infected plants leads to several abnormalities like stunting, curling of leaves, abnormal floral and root structure, callous like outgrowths in plants, this indicates a regulation of geminiviral response by abscisic acid [144]. ABA is known to enhance the survival capability of plants in drought conditions. Recently, an interesting finding described enhanced drought tolerance capacity of transgenic *A. thaliana* plants expressing TYLCV C4 protein [131]. However, this alteration of the physiological aspect of infected plants is through ABA independent mechanism.

Ethylene (ET) is another plant hormone that is involved in defence mechanism. The level of 1-aminocyclopropane-1-carboxylate oxidase (ACCO), a vital molecule of ethylene biosynthesis pathway, is increased during TYLCSV and CaLCV infection. However, 1-aminocyclopropane-1-carboxylate synthase 8 (ACS8) that catalyses the rate-limiting step in the biosynthetic pathway of ethylene is downregulated during TYLCSV infection [67]. The level of ACS8 targeting miR159 increases along with the disease progression, reducing the level of 1-aminocyclopropane-1 carboxylic acid (ACC), another ethylene precursor, which is probably compensated by the upregulation of ACCO. A dynamic equilibrium involving ET signalling plays at the interface of host and virus interaction [67]. Ethylene responsive factor 1 (ERF1) gene, the regulator of ethylene-responsive genes was also upregulated when ACMV TrAP protein was overexpressed in *N. tabacum* [145]. Likewise, the systemic silencing of CONSTITUTIVE TRIPLE RESPONSE 1 (CTR1), a negative regulator of ET signaling enhances upregulation of defence marker genes during tomato leaf curl Joydebpur virus (ToLCJoV) infection [146]. Suppressed expression of essential ET responsive EIN2 in case of WDV infecting a monocot plant might underline the evolutionary diversification of the plants [142].

BCTV C4 protein induces severe development abnormalities like hyperplasia of phloem tissue and tumour-like outgrowths in infected plants, and conversely, a mutation in C4 causes reduction in disease symptoms [147]. When brassinosteroids and abscisic acid were applied exogenously, the C4 transgene-induced phenotype of seedlings was partially rescued. However, seedlings became more sensitive to gibberellic acid and kinetin [148]. An earlier report revealed that *Arabidopsis* Shaggy-like kinase proteins (AtSKs), which targets transcription factors that regulate brassinosteroid signalling also interact with the C4 protein of BCTV and TGMV [149]. Current transcriptomic studies in response to the *WDV* in *Triticum aestivum* showed differential expression of BR signalling genes [142]. Different classic and stress-responsive phytohormones act in concert in the plants’ immunity and it is important to decipher the roles of these hormones in this complex network.

### Metabolite interplay

Plants varied responses against biotic stresses are often associated with the production of a variety of metabolites. Beet mild curly top virus (BMCTV) infection on chilli pepper induces a high level of glucose and fructose, galactose, and myoinositol compared to asymptomatic samples [150]. Glucose and fructose act as energy sources for running viral machinery, while galactose may be required for the synthesis of the glycoprotein required for capsid formation [151]. As an osmoregulator, myoinositol is also involved in tissue deformation during ageratum enation virus (AEV) infection [152]. In chilli, geminivirus infection induces prominent symptoms of leaf curling, yellowing, etc., and reduces the total chlorophyll a and b content affecting the CO_2_ fixation rate and total soluble sugars, proteins and starch content [6, 153–155]. TYLCV infection increases total phenolics, tannins, and the related gene expression but reduces the soluble sugars and free amino acids that impacts the growth and fecundity of whitefly [156]. Alteration of nutritional changes brought about by geminiviruses favour the abundance, fecundity, and transmission ability of vector whitefly to promote the spread of the virus. Various volatile organic substances released from secretory organs such as glandular trichomes, secretory cavities, and resin ducts, specifically acts as an attractant or repellent to specific herbivores and insect vectors. A fatty acid derivative undecanone, sesquiterpene zingiberene, and its transformed form, curcumene produced from tomato plants are reported to be toxic to whitefly [157]. Resistance towards one of the whitefly species was observed when zingiberene containing ginger oil was applied on the leaves of the tomato plant [158]. P-cymene, one of the active and toxic volatile substances may also play a role in repelling whitefly [157]. The infestation of whitefly leads to the upregulation of terpenoid biosynthesis genes. This secondary metabolite mediated defence is compromised by viral infection as the virus attenuates the terpenoid release. The number of whiteflies in different development stages were also higher in plants with silenced 5-epi-aristolochene synthase (EAS) gene, a terpenoid synthesis gene in tobacco [159]. βC1 protein encoded from TYLCCNB associated with TYLCCNV inhibits terpene synthesis by interacting with MYC2 transcription factor [138]. Utilizing the chemistry of secondary metabolites in controlling the herbivory can be an easy and time-efficient approach in managing the whitefly populations in field conditions.

### Innate genetic factors and hypersensitive response mediated responses

Disease resistance to phytopathogens is classified into nonhost resistance and host resistance. In the context of viral pathogenesis, nonhost resistance is a species-dependent phenomenon where the genotypes belonging to particular plant species might exhibit resistance or susceptibility to a specific virus [160]. While in the case of host resistance, it is typically limited to specific genotypes or cultivars of same or different species and renders the rest of them to be susceptible to the virus infection. There are multiple genetic studies describing the disease resistance phenotype is explicitly associated with occurrence of gene loci encode for resistance (R) genes. In the dynamic population, R-genes segregate into dominant and recessive. Often it is found that the former exerts defence responses mostly by induction of HR response [161] and the latter inhibits the virus life cycle by impeding the protein translation [162]**.** Identification of promising resistant genetic sources from domestic and wild varieties has been a longstanding successful approach in managing the geminiviruses against several crops such as tomato, bhendi, cassava, cotton and mung bean [163].

Frequent occurrence of TYLCV epidemics in tomato cultivating regions has posited the tomato infecting geminivirus such as TYLCV as a potential threat for the production. Genetic approaches to gain tolerance/resistance to TYLCV resulted in the mapping of six resistance Ty- loci, i.e. *Ty* 1–6 from different wild tomato species and, except for *Ty-4* and *Ty-6,* rest of the *Ty* genes have been characterized (Table 1). *Ty* genes confer phenotypic disease tolerance to begomoviruses, but unlike the reported R-genes, doesn’t induce HR. Tomato varieties possessing Ty-1/Ty-3 alleles upon infection with TYLCV produced mild or no symptoms with low virus titre, but an increased accumulation of siRNAs. Ty-1 and Ty-3 are allelic forms of ϒ type of RNA dependent RNA polymerase gene (RDRϒ) [164]. As increased siRNAs production derived from V1 and C3 genes, enhanced TGS conferred Ty-1 mediated resistance in TYLCV infected plants where hypermethylation of cytosine residues in the V1 and C3 promoters of tomato severe rugose virus (ToSRV) were observed [165]. However, the resistance mediated by *Ty-1* against TYLCV was compromised by mixed infection with cucumber mosaic virus (CMV). CMV encodes silencing suppressor proteins which counteract the host RNAi machinery and trade-off TYLCV resistance in plants and enhances the viral titre and infection severity [165]. Ty-1/Ty-3 have been found to be essential for achieving broad-range resistance against geminiviruses as they provide a high degree of resistance to both mono and bipartite begomoviruses [166]. *Ty-2* is a functional R gene that encodes for nucleotide-binding-leucine rich repeat protein. The insertion of *Ty-2* gene into the domestic susceptible tomato plants conferred resistance to TYLCV [167]. Quantitative trait locus, *Ty-5* majorly has also been implicated in recessive resistance against TYLCV in TY172 line of tomato [168]. At this locus, *Pelo* gene which encodes for mRNA surveillance factor pelota (pelo) homolog in tomato, is involved in ribosome recycling phase of protein biosynthesis and controls the disease resistance [162]. Experimental evidence suggests that *Pelo* silenced susceptible transgenic plants infected with TYLCV failed to produce disease symptoms, and viral titre was decreased by 20–60-fold. The possible mechanism might involve affected ribosome dissociation, leading to low availability of ribosomal subunits for translation initiation of viral proteins [162]. Ty-4 and Ty-6 also provide resistance against TYLCV and tomato mottle virus (ToMV) [169, 170]. Apart from exploiting *Ty* loci in breeding, devising the molecular biology tools may provide an opportunity for developing broad resistance against plant viruses. However, the resistance conferred by Ty genes can be compromised by the presence of betasatellite during the infection [171]. Breeding approaches also have identified inter simple sequence repeat (ISSR), a key diagnostic marker, in ToLCNDV tolerant cultivar *Solanum habrochaites* LA1777 which can be exploited for marker-assisted breeding in rising defence against ToLCNDV. Two genetic markers SSR18_170-145_ and SSR304_158-186_ have been identified from the F2 population of susceptible variety Punjab Chhuhara (PBC), ‘H-24’, and *S. habrochaites* accession ‘EC-520061’ with possible implications in TYLCV resistance [172].

Three resistance genes CMD1, CMD2 and CMD3 have been identified against cassava mosaic geminiviruses that are prevalent in South Africa and India. CMD1 is a polygenic resistance gene originally from *Manihot glaziovii* [173]. Several CMD resistant varieties were obtained utilizing CMD1 through breeding that exhibited lower viral titre than the susceptible ones and had reduced systemic movement of virus enabling to develop virus-free plants from infected cuttings [174]. Molecular genetic mapping and analysis have led to the identification of CMD2, a monogenic dominant locus from *M. esculenta*. Crossing CMD1 and CMD2 carrying parents, that together produce complementary resistant effect, have generated CMD3, another quantitative trait loci responsible for resistance in cassava [175]. Although several breeding programs successfully obtained CMD resistant varieties, molecular characterization of these genes has not yet progressed much. As geminiviruses can evolve into more virulent strains and break the natural resistance provided by marker-assisted selection and breeding, it is important to develop resistance against virus by additional genetic engineering methods.

Because of its high-quality fibre and superior lint *G. hirsutum* accounts for more than 90% of total cotton production all over the world. But it is highly susceptible to cotton leaf curl disease (CuLCD) caused by Cotton leaf curl virus [176]. *G. arboreum*, one of the wild progenitors of *G. hirsutum,* has been highly tolerant to various biotic and abiotic stresses and a major source of genes for natural resistance. Through introgression and conventional hybridization programs, single genes with dominant effect for resistance were transferred from *G. arboreum* to *G. hirsutum* [177]. Massive screening of 22 cotton varieties revealed two genes R_1CLCuDhir_ and R_2CLCuDhir_ that were involved in *G. hirsutum* resistance and one gene S_CLCuDhir_ as suppressor of resistance [178]. However, introgression of multiple genes for resistance with minor effects can provide plants with durable resistance [179].

Bhendi (*Abelmoschus esculentus)* is an important vegetable crop in the tropical and subtropical countries of Indian subcontinent is greatly challenged by bhendi yellow vein mosaic virus (BYVMV) and okra enation leaf curl virus (OELCV). Various bhendi resistant varieties have been developed through conventional breeding experiments in the past 50 years. The responsible factors of natural resistance transferred during the breeding were either two recessive genes or two complementary dominant genes [180–184]. Presence of a single dominant gene or two dominant genes may also provide resistance against the virus [185–187] (Table 2). Various molecular markers RAPD, SSR, AFLP, have been seemingly associated with the resistant genes identified which may assist in characterizing resistance genes.

**Table 2:** Sources of resistance genes and exploitation of wild varieties for developing resistance to geminiviruses

Host	Genetic factor	Encoded protein	Source	Target geminivirus	References
Tomato	*Ty-1*	RDRγ	Chromosome 6 of *S. chilense*	Tomato yellow leaf curl virus (TYLCV), honey suckle yellow vein mosaic virus (HYVMV) and tobacco leaf curl Japan virus (TbLCJV)	[148]
	*Ty-2*	NBS-LRR	Chromosome 11 of *S. habrochaites*	TYLCV	[151]
	*Ty-3*	RDRγ	Chromosome 6 of *S. chilense*	TYLCV	[148, 150]
	*Ty-4*	Uncharacterized	Chromosome 3 of *S. chilense*	TYLCV	[154]
	*Ty-5*	Pelota	Chromosome 4 of *S. chilense*	TYLCV	[146]
	*Ty-6*	Uncharacterized	Chromosome 10 of *S. chilense*	TYLCV	[153]
	Inter simple sequence repeat (ISSR) Marker	–	*S. habrochaites*	Tomato leaf curl New Delhi virus (ToLCNDV)	[203]
	SSR304_158–186_ and SSR18_170–145_	–	Chromosome 7 and 10 of *Solanum habrochaites* accession ‘EC-520061	Tomato leaf curl virus (ToLCV)	[156]
Cassava	CMD1, recessive gene and polygenic	Uncharacterized	*Manihot glaziovii* (TMS)	African cassava mosaic virus (ACMV), East African cassava mosaic virus (EAMCV)	[157]
	CMD2, a major dominant gene	Uncharacterized	Chromosome 12 of *M. esculenta* (TME)	ACMV, EAMCV	[159]
	CMD3, a QTL	Uncharacterized	TMS 97/2205	ACMV, EAMCV	[204]
Okra	Two duplicate dominant genes and two complementary genes	Uncharacterized	BCO-1 and VNR Green	Bhendi yellow vein mosaic virus (BYVMV)	[164]
	Two dominant genes	Uncharacterized	*A. manihot* ssp. *Manihot*	BYVMV	[170]
	Two recessive genes	Uncharacterized	Pusa Sawani (IC-1542 X Pusa Makhmali)	BYVMV	[165]
	Two complementary dominant genes	Uncharacterized	*A. manihot* (L.) Medikus ssp. manihot	BYVMV	[167]
	Two complementary dominant genes and two duplicate dominant genes	Uncharacterized	Arka Anamika, Punjab Padmini and Arka Abhay	BYVMV	[168]
	Single dominant gene	Uncharacterized	*A. manihot*	BYVMV	[171]
	Two dominant complementary genes	Uncharacterized	*A. manihot* ssp*. Manihot*	BYVMV	[166]
	A single dominant gene (Not Identified)	Uncharacterized	*A. manihot* (L.) *Medik* and *A. manihot* (L.) *Medik* ssp. *Manihot*	BYVMV	[169]
Black gram	*CYR1,* a dominant gene	Non-TIR-NBS-LRR class R-gene	*V. mungo* line VM1 (MYMIV resistant)	Mung bean yellow mosaic virus (MYMV)	[205]
Common bean	*PvVTT1,* a dominant gene	Protein with a double TIR motif	*Phaseolus vulgaris* cv. Othello	Bean dwarf mosaic virus (BDMV)	[145]
	*bgm-1,* a recessive gene	Uncharacterized	Chromosome 5 of DOR476 or Dry bean landrace cultivar Garrapato (Mexico)	Bean golden mosaic virus (BGMV)	[172]
	*bgm-2,* a recessive gene	Uncharacterized	DOR303	BGMV	[173]
	*Bct-1,* a dominant gene	Uncharacterized	Chromosome 7 of *P.* coccineus Burtner	Beet curly top virus (BCTV)	[176]
	*Bgp-1,* a dominant gene	Uncharacterized	PR9556-171	BGMV	[174]
	*PvBlc,* a dominant gene	Uncharacterized	*P. vulgaris* line GG12	TYLCV	[175]
Cotton	*R1CLCuDhir* and *R2CLCuDhir*	Uncharacterized	CP-15/2, LRA-5166, CIM-443, Ravi, Cedex	Cotton leaf curl Multan virus (CLCuMV), cotton leaf curl Kokhran virus	[162]
Melon	A major QTL located in chromosome 11, and two other locus on chromosome 2 and 12	–	*Cucumis melo* subsp*. Agrestis* (WM-7)	ToLCNDV	[179]
	A single recessive gene	Uncharacterized	Chromosome 8 of *Cucurbita moschata*	ToLCNDV	[180]
Sponge gourd	A single dominant gene	Uncharacterized	DSG-6 and DSG-7	ToLCNDV	[177]
	A single dominant gene and two sequence-related amplified polymorphism (SRAP) markers	Uncharacterized	DSG-6	ToLCNDV	[178]

Responsible for a yield loss of up to 100%, *Bean golden mosaic virus* is one of the major concerns for common bean (*Phaseolus vulgaris*) production. Resistant recessive genes, *bgm-1* and *bgm-2* were identified from a highly disease-resistant variety of bean from Mexico [188, 189]. Later on, several durable breeding lines were developed utilizing these resistance genes. Another R gene from *Phaseolus vulgaris* cultivar Othello, named *PvVTT1* (*Phaseolus vulgaris* VIRUS response TIR-TIR GENE 1) found to be responsible for resistance against bean dwarf mosaic virus (BDMV) through HR mediated defence response [161]. The dominant resistance gene *Bgp-1* has also been reported to account for normal pod development during viral infection and involve in providing resistance against BGYMV [190]. Similarly, bean leaf crumple disease  of  *P. vulgaris* is associated with TYLCV and is controlled by the dominant gene *PvBlc* [191]. *Phaseolus* also has resistance gene *Bct-1*, linked to the RAPD marker against BCTV [192]. In addition, the expansion of genetic studies on cucurbits against ToLCNDV infection revealed the occurrence of a single dominant resistance gene in *Luffa cylindrica* (Roem.) [193, 194]. Three candidate genes [195] and recently, one major quantitative trait locus (QTL) on chromosome 8 conferring resistance to ToLCNDV have been found in melon [196].

Natural hybridization and conventional breeding programs have utilized the natural resistance resources of plant genomes to combat virus infection via introgression for successful management of plant viruses. However, to attain stable resistance, it is important to further analyse and characterize these genetic sources and implementation of advanced speed breeding techniques for advanced phenotyping and quick transformation outputs. Although findings are promising in controlling the geminiviruses, resistance breakdown occurs frequently requiring continuous exploration of new resistance sources among geminivirus infecting crops. As geminiviruses have been observed in association with nanovirus, potyvirus, and other plant RNA viruses, investigation of commonly conserved genetic factors among the phytopathogens is crucial for tackling the mixed infections in regular filed conditions [197]. For instance**,** the phenomenon of single R-gene controls the unrelated pathogen clusters. Wheat *Bdv1* locus shows resistance to barley yellow dwarf virus (BYDV) also interfere R-genes of fungal pathogens, causing leaf rust [198].

Plant immune system has evolved multi-layer receptor systems to sense and induce the pathogen defence responses. The first layer of defence employs the cell surface radars known as pathogen recognition receptors (PRR) that recognise extracellular immune targets called pathogen associated molecular patterns (PAMPs). However, to circumvent this basal defence, pathogens employ sophisticated intracellular immune suppressors called effectors (Avr) which in turn are intercepted by the host resistance (R) genes, that encode for NBS-LRR type receptors, leading to rapid immune responses called effector-triggered immunity (ETI) [199]. These R-genes sense the effectors via direct or indirect interactions to initiate physiological and biochemical defence responses such as reactive oxygen species (ROS) generation, cell wall fortification, defence gene expression and HR at the infection site followed by induced SAR, at the distant leaves to restrict the pathogen growth and systemic movement [199]. Bean dwarf mosaic virus (BDMV)-NSP elicits HR response in BDMV resistant bean cultivar Pinto bean cvs. Othello [200]. Similarly, Rep protein of ACMV and TYLCV, pre-coat protein of tomato leaf curl Java virus (ToLCJV), and TrAP of TYLCSV also induce HR, so also does the recently identified a novel TYLCCNB βV1 gene [19, 201–203]. Often, overexpression of individual geminiviral ORFs induces visible HR or necrotic symptoms, which do not appear during virus infection, suggesting the geminiviruses also encode for proteins that mediate HR suppression. AC4 protein of ACMV may negatively affect the Rep mediated HR responses to promote virulence [201] while TLCYnV encoded C4 physically interacts with HIR1 (hypersensitive induced reaction 1) and perturbs the HIR1 homodimerisation to counteract HIR mediated HR response [204]. Due to limited coding capacity, unlike other plant pathogens, viruses do not strictly fit into the Gene-for-Gene theory of host–pathogen interactions. However, to overcome the limitation, viruses have evolved multifunctional proteins that maintain a coevolutionary relationship with the host to invade the host defence machinery.

### Other cellular factors

The chloroplast is a hub of plant’s immune arsenal, not only by producing immune signals such as ROS and SA but also to initiate the biosynthesis pathways of the phytohormones GA, ABA and JA that are crucial for biotic and abiotic stress tolerance [205]. Hence, to suppress the active host defence, viruses primarily target the chloroplast machinery to attain successful infection [206]. Radish leaf curl betasatellite (RaLCB) encoded βC1 protein disturbs chloroplast organization, photosynthetic efficiency and causes veinal chlorosis [6]. A recent finding suggests PsbP (photosystem II subunit P), an extrinsic protein of oxygen-evolving complex (OEC), plays a defensive role against geminiviruses [207]. *PsbP-*silenced plants, had higher virus titre  than the control plants and *PsbP* overexpression lines showed reduced disease symptoms evidenced by lower virus replication at early stages of infection. However, during later phases of infection, RaLCB-βC1 interacts with PsbP and permits successful virus replication [207]. Host regulatory proteins such as GRAB (Geminivirus Rep-A binding) proteins of NAC transcription factor families, that are involved in leaf development and senescence, can be modulated to interacting with viral proteins such as RepA to facilitate viral replication or suppress host immunity [208]. Interaction with GRAB protein has been shown to inhibit WDV infection, but enhance TYLCSV infection [209]. Overexpression of *Arabidopsis* TIFY4B, a plant DNA binding protein, responsible for cell cycle arrest, causes a reduction in viral titre and increase the latent period for symptom production. Increased expression of TIFY4B after geminiviral infection also suggests its crucial role in defence against the viruses. Furthermore, to hinder the plant defence response, TrAP protein encoded by TGMV and CaLCuV interacts with TIFY4B to counteract the cell cycle arrest leading to increased viral load [210].

### CRISPR-Cas9 based resistance against geminivirus

The application of advanced genetic engineering methods has helped to overcome the limitations of labour-intensive traditional approaches for developing resistant plants. CRISPR-Cas (Clustered, regularly interspaced short palindromic repeats-CRISPR associated protein) is a bacterial adaptive immune strategy against invasive foreign nucleic acids, that has been exploited to target plant viruses. In this technique, single guide RNA (sgRNA) directs an endonuclease Cas9 to modify specified viral DNA targets by inducing double-stranded breaks which eventually leads to viral genome degradation. This technique has already been proved successful in reducing the viral titer and symptom expression against the monopartite and bipartite geminiviruses [211]. Transient expression of sgRNA-Cas9 directed to dsDNA intermediate forms of BSCTV has reduced the viral accumulation by 80% and abolished the disease symptoms in *N. benthamiana* plants [211]. The findings of Baltes et al. [212] corroborated the CRISPR system's efficiency against bean yellow dwarf virus (BeYDV)-eGFP by estimating the fluorescence intensity relative to the control plants. Systemic targeting of BeYDV key regulatory elements such as conserved nonanucleotide hairpin, rep binding sites (RBS), and Rep protein motifs that are crucial for replication has reduced the viral DNA accumulation [212]. Considering the sequence-specificity of the technique, targeting the conserved geminiviral IR region with a suitable a sgRNA (IR-sgRNA) can provide a universal approach to control geminivirus infections, even for the commonly occurring mixed infections in the field conditions. Systemic delivery of IR-sgRNA in *N. benthamiana* Cas9OE plants infected separately with TYLCV, BCTV, and merremia mosaic virus (MeMV) displayed symptom attenuation and suppression of viral DNA against infections [213]. Notwithstanding the promising CRISPR applications, CRISPR-induced virus evolution must be critically examined to monitor the emergence of resistant viruses [214]. Recently the breakdown of CRISPR resistance has been reported, where ACMV-AC2 evolved a conserved single "T" insertion that can affect the Cas9 target-cleavage activity [215]. Additionally, non-homologous end joining (NHEJ) repair of plant system is a posing threat that could repair the Cas9 targeted viral dsDNA intermediates [213, 215]. Importantly recombination, a driving force of geminivirus evolution, which allows swapping of genomic sequences during mixed infections, also enables the geminiviruses to escape the CRISPR induced cleavage, subsequently leading to CRISPR resistance.

## Conclusions

Plants have evolved to develop very complex defence strategies against geminiviral infection. RNA silencing machinery remains to be one of the prominent mechanisms. While TGS carries out viral genome methylation, consequently, leads to the repression of viral pathogenicity proteins, PTGS mediates the degradation of the viral mRNAs, thereby inhibit the viral infection. Several chromatin remodelers have evolved in plants that carry out repressive modifications on host genome, diverts targeting viral genome. miRNAs have emerged as effective tools for achieving broad spectrum resistance against geminiviruses. Another level of defence against geminiviruses is mediated through R-genes that are well studied in case of fungi and bacteria and in this context geminiviruses are highlighted recently. Several other host defence regulatory mechanisms like autophagy, ubiquitination, hormonal signalling, protein kinases also play a significant role in guarding and shielding the host from geminivirus by providing the ammunition to the host to act against geminivirus. Against these wide array of defence mechanisms, various suppressor proteins and evolved sophisticated strategies are deployed by the geminiviruses that emphasize the dynamic relationship between the host and the pathogens and unique role of geminiviruses in driving the co-evolution of both plants along with their own. Molecular studies carried out to elucidate the antiviral responses involve the characterization of potential targets in cellular transcriptome, proteome, metabolome in the background of geminivirus interaction. In the vast array of cellular pathways, identifying the mechanisms which does not influence the plant growth remains the principal task. Current efforts focus on the use of precise gene-editing tool for providing a broad range of resistance against viruses. Various laboratories worldwide are standardizing the CRISPR-cas9 system for providing broad range of adaptive immunity and resistance against geminiviruses. Selection of targets within viral genome is crucial factor in achieving the durable resistance. In this context, non-coding targets are more efficient over coding regions as they embed the crucial elements essential for virus replication and pathogenicity maintenance [216]. Incidences of geminiviral diseases are increasing at an accelerated pace due to high evolution rate expanding their geographical barrier and host range. Although, various techniques ranging from conventional methods to molecular approaches have been adopted to control the geminiviral infections, due to mixed virus infections the success is limited. Identifying the suitable host factors involved in the resistance during plant-geminivirus interaction, the introduction of multiplexed genetic engineering tools targeting multiple targets, and targeted deletion of large sequences from the viral genomes can aid in the development of disease-free plants and preventing the emergence of CRISPR resistant geminiviruses [217, 218].

## Data Availability

Not applicable.

## Abbreviations

CR: Common region
REP: Replication-associated protein
TrAP: Transcription activator protein
REn: Replication enhancer protein
CP: Coat protein
MP: Movement protein
NSP: Nuclear shuttle protein
TGS: Transcriptional gene silencing
PTGS: Post-transcriptional gene silencing
HR: Hypersensitivity response
RDR2: RNA- DEPENDENT RNA POLYMERASE 2
CLSY1: CLASSY 1
DCL-3: DICER-LIKE 3
HEN1: HUA- ENHANCER 1
AGO4: ARGONAUTE 4
RISC: RNA-induced silencing complex
BCTV: Beet curly top virus
DDR complex: DEFECTIVE IN RNA DIRECTED DNA METHYLATION 1 (DRD1), DEFECTIVE IN MERISTEM SILENCING 3 (DMS3), REQUIRED FOR DNA METHYLATION 1 (RDM1) complex
NPRE1: Nuclear RNA polymerase E
KTF1: KOW DOMAIN-CONTAINING TRANSCRIPTION FACTOR 1
DRDM2: DOMAINS REARRANGED METHYL TRANSFERASE 2
SUVH4: SU(VAR)3–9 HOMOLOGUE 4
KYP: KRYPTONITE
CMT3: CHROMOMETHYLTRANSFERASE 3
RAV: RELATED TO ABI3 and VP1
DDM1: Decrease in DNA methylation 1
Adk1: ADENOSINE KINASE 1
TYLCV: Tomato yellow leaf curl virus
BSCTV: Beet severe curly top virus
SAMDC1: S-adenosyl-methionine decarboxylase
SAM: S-Adenosyl-methionine
dSAM: Decarboxylated SAM
SAHH: S-adenosyl homocysteine hydrolase
MET1: Methyltransferase 1
HDA6: Histone deacetylase 6
EML1: EMSY-LIKE 1
RPT4a: REGULATORY PARTICLE TRIPLE-A ATPASE 4A
ToLCNDV: Tomato leaf curl New Delhi virus
RDR1: RNA-dependent RNA polymerase 1
ToLCGV: Tomato leaf curl Gujarat virus
VMYMV: Vigna mungo yellow mosaic virus
DCL: DICER- LIKE protein
DRB: DsRNA binding proteins
AS2: ASYMMETRIC LEAVES 2
DCP2: DECAPPING 2
CaM: Calmodulin-like protein
SGS3: SUPPRESSOR OF GENE SILENCING 3
WDV: Wheat dwarf virus
CLCuMuB: Cotton leaf curl Multan betasatellite
TMV: Tobacco mosaic virus
ToLCV: Tomato leaf curl virus
MYMIV: Mung bean yellow mosaic India virus
MYMV: Mungbean yellow mosaic virus
CRLs: Cullin ring ligases
SCF complex: SKP1 (S phase kinase-associated protein 1)-CUL1(CULLIN 1)-F-box-protein
CSN: COP9 Signalosome
COI1: CORONATIN INSENSITIVE 1
JA: Jasmonic acid
JA-Ile: Jasmonoyl isoleucine
JAZ: Jasmonate Zim domain
NtRFP1: Nicotiana tabacum RING FINGER PROTEIN 1
UBA1: Ubiquitin activating enzyme
UBC3: Ubiquitin-conjugating enzyme 3
CKIs: Cyclin-dependent kinase inhibitors
KPC: Kip-1 ubiquitination promoting complex
RKP1: Ring finger protein
UPS: Ubiquitin-proteasomal system
SCE1: SUMO CONJUGATING ENZYME 1A
TGMV: Tomato golden mosaic virus
TYLCSV: Tomato yellow leaf curl Sardinia virus
ACMV: African cassava mosaic virus
PCNA: Proliferating cell nuclear antigen
ATG8: AUTOPHAGY-RELATED 8
CLCuMuV: Cotton leaf curl Multan virus
TLCYnV: Tomato leaf curl Yunnan virus
TYLCCNB: Tomato yellow leaf curl China betasatellite
RLKs: Receptor like kinases
SnRK1: SUCROSE NON-FERMENTING1 related protein kinase 1
CaLCuV: Cabbage leaf curl virus
GRIK1: Geminivirus Rep interacting kinase 1
AtREM4: Arabidopsis thaliana remorin group 4
ATP: Adenosine triphosphate
AMP: Adenosine monophosphate
MAPKs: Mitogen-activated protein kinase
SA: Salicylic acid
PR: Pathogenesis-related
MKK2: Map kinase kinase 2
PRRs: Pattern recognition receptors
MAMPs: Microbe-associated molecular patterns
NIK: NSP interacting kinase
RPL10: Ribosomal protein 10
LIMYB: L10-interacting MYB domain-containing protein
CLV1: CLAVATA 1
BAM1: BARELY ANY MERISTEM 1
AtBIN2: Arabidopsis thaliana Brassinosteroid insensitive 2
SPLCV: Sweet potato leaf curl virus
FLS2: FLAGELLIN SENSING 2
BRI1: BRASSINOSTEROID INSENSITIVE 1
PERK: Proline-rich extension-like receptor kinase
TCrLYV: Tomato crinkle leaf yellows virus
PXYs: PHLOEM INTERCALATED WITH XYLEM members
PEPRs: PEP1 RECEPTOR members
GDU3: GLUTAMINE DUMPER 3
SOD: Superoxide dismutase
POD: Peroxidase
CAT: Catalase
MeJA: Methyl jasmonate
JAI1: JASMONATE INSENSITIVE 1
PDF1.2: PLANT DEFENSIN 1.2
CORI3: CORONATINE INSENSITIVE 13
GA: Gibberellic acid
GAI: Gibberellic-acid insensitive
SAUR: Small auxin upregulated RNA
AUX: Auxin/Indole-3-acetic acid
ORR22: Oryzae sativa response regulator
SCTV: Spinach curly top virus
ATHB7: Arabisopsis thaliana homeobox 7
ET: Ethylene
ACCO: 1-Aminocyclopropane-1-carboxylate oxidase
ACS8: 1-Aminocyclopropane-1-carboxylate synthase 8
ACC: 1-Aminocyclopropane-1 carboxylic acid
ERF1: Ethylene responsive factor 1
CTR1: CONSTITUTIVE TRIPLE RESPONSE 1
ToLCJoV: Tomato leaf curl Joydebpur virus
BMCTV: Beet mild curly top virus
AEV: Ageratum enation virus
EAS: 5-Epi-aristolochene synthase
RDR: RNA dependent RNA polymerase
ToSRV: Tomato severe rugose virus
CMV: Cucumber mosaic virus
ToMV: Tomato mottle virus
ISSR: Inter simple sequence repeat
CMD: Cassava mosaic disease
CuLCD: Cotton leaf curl disease
CuLCV: Cotton leaf curl virus
BYVMV: Bhendi yellow vein mosaic virus
OELCV: Okra enation leaf curl virus
RAPD: Random amplified polymorphic DNA
AFLP: Amplified fragment length polymorphism
BGMV: Bean golden mosaic virus
BDMV: Bean dwarf mosaic virus
BYDV: Barley yellow dwarf virus
NBS-LRR: Nucleotide-binding site and leucine-rich repeat
ETI: Effector-triggered immunity
SAR: Systemic acquired resistance
ToLCJV: Tomato leaf curl Java virus
HIR1: Hypersensitive induced reaction 1
RaLCB: Radish leaf curl betasatellite
PsbP: Photosystem II subunit P
OEC: Oxygen-evolving complex
GRAB: Geminivirus Rep-A binding
CRISPR-cas9: Clustered regularly interspaced short palindromic repeats and CRISPR-associated protein 9
NHEJ: Non-homologous end joining
